# Supplementary material for: Content-rich biological network constructed by mining PubMed abstracts
Source: BMC Bioinformatics. 2004 Oct 8;5:147. doi: 10.1186/1471-2105-5-147 (PMC528731; doi:10.1186/1471-2105-5-147)
Supplement: Additional File 2 — The original results of the above study (non-essential files are deleted to keep the file size under the limit set by BMC bioinformatics). [file 1471-2105-5-147-S2.bz2 › chilibotAdditionalFile2/dip40/365ID8663044E6038/html/FGFR2_FGF7.html]

 


 **FGFR2** and **FGF7** 
  
Found 200 abstracts in PubMed, retrieved 40.  
 

 What does Google say? 
 PDF only 
| .edu only 

---

**Interactive relationship** (e.g. stimulation, inhibition, etc)

**Stimulatory relationship**- RESULTS Although KGF  [ **FGF7** ]  is known to target only epithelial cells, all of the three cell lines transduced with K sam  [ **FGFR2** ]  proliferated due to KGF  [ **FGF7** ]  stimulation.  Ref: 11937263 Exp Hematol, 2002
- Both are mainly synthesized by mesenchymal cells and stimulate epithelial cells via KGF  [ **FGF7** ]  receptor KGFR  [ **FGFR2** ]  which is a splice variant of FGFR 2  [ **FGFR2** ] .  Ref: 12565793 Exp Mol Pathol, 2002
- KGF  [ **FGF7** ]  treatment significantly increased production of CFCs derived from K sam  [ **FGFR2** ]  transduced bone marrow cells without causing the exhaustion of immature CFCs.  Ref: 11937263 Exp Hematol, 2002
- Keratinocyte growth factor  [ **FGF7** ]  stimulates the growth and activity of epithelial cells via the keratinocyte growth factor  [ **FGF7** ]  receptor  [ **FGFR2** ] .  Ref: 12351666 J Dent Res, 2002
**Neutral relationship**- **FGF7**  and FGF10 activate only the b isoform of  **FGFR2**  FGFR2b.  Ref: 12591959 Proc Natl Acad Sci U S A, 2003
- A lymphoid cell line BaF KGFR  [ **FGFR2** ]  containing the FGF 7  [ **FGF7** ]  receptor  **FGFR2**  IIIb was treated with FGF 7  [ **FGF7** ]  and with various glycosaminoglycans.  Ref: 12215437 J Biol Chem, 2002
- KGF  [ **FGF7** ]  upregulated EC KGFR  [ **FGFR2** ]  mRNA expression, predominately in the crypt and lower portion of the villus.  Ref: 12757114 JPEN J Parenter Enteral Nutr< MedlineTA>JPEN J Parenter Enteral Nutr, 2002
- Rat kidney organ culture experiments in the presence of a soluble  **FGFR2**  IIIb chimera or a neutralizing antibody to  **FGF7**  suggested an important contribution of FGFs other than  **FGF7**  to the branching program.  Ref: 11731227 Mech Dev, 2001
- These findings suggest that the keratinocyte growth factor  [ **FGF7** ]  receptor  [ **FGFR2** ]  pathway of mesenchymal epithelial interaction could play an important part in the molecular pathogenesis of gingival hyperplasia.  Ref: 12351666 J Dent Res, 2002
- Keratinocyte growth factor  [ **FGF7** ]  receptor  [ **FGFR2** ]  KGFR  [ **FGFR2** ] , also known as fibroblast growth factor receptor FGFR 2  [ **FGFR2** ]  IIIb, is located in many types of epithelial cells and is activated by four known ligands FGF 1, FGF 3, FGF 7  [ **FGF7** ]  also known as KGF  [ **FGF7** ]  and FGF 10 that are predominantly synthesized by mesenchymal cells.  Ref: 12608893 Pathol Int, 2003
- Exposure to both UVB 30 150 mJ cm 2 and CUH 200 microM of NIH3T3 KGFR  [ **FGFR2** ]  KGF  [ **FGF7** ]  receptors transfectants caused a rapid tyrosine phosphorylation and activation of KGFR  [ **FGFR2** ]  similar to that induced by KGF  [ **FGF7** ] , and internalization of the activated receptor.  Ref: 12717419 Oncogene, 2003
- CONCLUSIONS KGF  [ **FGF7** ]  could efficiently induce proliferation of hematopoietic cells expressing the K sam  [ **FGFR2** ]  gene without obvious induction of differentiation or exhaustion of immature progenitor cells.  Ref: 11937263 Exp Hematol, 2002
- Keratinocyte growth factor  [ **FGF7** ]  regulates proliferation and differentiation of hematopoietic cells expressing the receptor gene K sam  [ **FGFR2** ] .  Ref: 11937263 Exp Hematol, 2002
- The relative mitogenic efficacy of basic FGF, acidic FGF, or KGF  [ **FGF7** ]  was in correlation with their affinities for the KGF  [ **FGF7** ]  receptor KGFR  [ **FGFR2** ] .  Ref: 12761878 J Cell Biochem, 2003
- Taken together our results demonstrate that exposure to UVB and to oxidant stimuli induces a rapid intracellular production of ROS, which in turn are capable of triggering KGFR  [ **FGFR2** ]  activation and internalization, similar to those induced by KGF  [ **FGF7** ] .  Ref: 12717419 Oncogene, 2003
- Dermatan sulfate also enabled FGF 7  [ **FGF7** ]  dependent phosphorylation of mitogen activated protein kinase and promoted binding of radiolabeled FGF 7  [ **FGF7** ]  to  **FGFR2**  IIIb.  Ref: 12215437 J Biol Chem, 2002
- It binds to and activates a receptor tyrosine kinase, the KGF  [ **FGF7** ]  receptor KGFR  [ **FGFR2** ] , which is a splicing variant of the fibroblast growth factor receptor 2  [ **FGFR2** ] .  Ref: 12122441 Histochem Cell Biol, 2002
- These findings suggest that FGF 7  [ **FGF7** ]  and FGF 10 contribute to the regeneration and differentiation of acinar cells and angiogenesis in AP through KGFR  [ **FGFR2** ] .  Ref: 12565793 Exp Mol Pathol, 2002
- Three stable cell lines expressing IIIb  **FGFR2**  demonstrated chemotaxis toward KGF  [ **FGF7** ] , but never proliferated, secreted urokinase, or formed tube like structure by KGF  [ **FGF7** ]  treatment.  Ref: 11735116 Biochem Biophys Res Commun, 2001
- Then we demonstrated that these effects of the mesenchyme were mimicked by fibroblast growth factor 7  [ **FGF7** ]   **FGF7** , a specific ligand of FGFR2IIIb, which is a tyrosine kinase receptor of the FGF receptor  [ **FGFR2** ]  family.  Ref: 11891329 Proc Natl Acad Sci U S A, 2002
- FGF1,  **FGF7** , FGF9, FGF10, FGF11, and FGF18 were also expressed in the pancreas at varying time points and levels, as well as FGF receptor  [ **FGFR2** ]  forms FGFR1b, FGFR1c, FGFR2b, FGFR2c, FGFR3b, and FGFR4.  Ref: 12666204 Dev Dyn, 2003
- Heparin and LMWH bind to low affinity sites on KGF  [ **FGF7** ]  and produce non specific inhibition, while KGFR2 beta IIIb Fc, a soluble chimera of an extracellular KGFR  [ **FGFR2** ]  fragment, is a more specific KGF  [ **FGF7** ]  inhibitor.  Ref: 12529961 Anticancer Res< MedlineTA>Anticancer Res, 2003

**Non-interactive relationship** (e.g. studied together, co-existance, homology, etc.)

- FGF 7  [ **FGF7** ]  did not support cell proliferation in the absence of glycosaminoglycan or with addition of heparan sulfate or chondroitin sulfate A C but did stimulate BaF KGFR  [ **FGFR2** ]  division in the presence of dermatan sulfate or highly sulfated low molecular weight fractions of dermatan.  Ref: 12215437 J Biol Chem, 2002
- The alpha5 integrin, keratinocyte growth factor  [ **FGF7** ]  receptor  [ **FGFR2** ]  and cyclin D1 mRNA levels were higher compared to the controls as early as 2 h after ethanol and 30 min after acetone treatment of the cells.  Ref: 12720008 Arch Dermatol Res< MedlineTA>Arch Dermatol Res, 2002
- Changes in the expression of KGF  [ **FGF7** ]  and KGFR  [ **FGFR2** ]  are less pronounced.  Ref: 11737224 Eur J Clin Invest, 2001
- RESULTS De novo AR expression by PC3 cells restores  **FGFR2**  IIIb isoform expression and sensitivity to  **FGF7**  and FGF2.  Ref: 12430142 Prostate, 2002
- Proliferation by KGF  [ **FGF7** ]  of murine primary bone marrow cells transduced with K sam  [ **FGFR2** ]  then was examined in liquid culture.  Ref: 11937263 Exp Hematol, 2002
- P63a mutations lead to aberrant splicing of keratinocyte growth factor  [ **FGF7** ]  receptor  [ **FGFR2** ]  in the Hay Wells Syndrome.  Ref: 12692135 J Biol Chem< MedlineTA>J Biol Chem, 2002
- Morphologic observation showed that KGF  [ **FGF7** ]  induced proliferation but did not cause significant differentiation of 32D K sam  [ **FGFR2** ]  cells.  Ref: 11937263 Exp Hematol, 2002
- Treatment with 5 OH 2 D 3 increased the levels of KGFR  [ **FGFR2** ]  in the presence but not in the absence of KGF  [ **FGF7** ] , probably due to inhibition of ligand induced receptor degradation.  Ref: 12761878 J Cell Biochem, 2003
- Our results indicate the possible involvement of both KGF  [ **FGF7** ]  and KGFR  [ **FGFR2** ]  in enhanced epithelial cell proliferative activity and recurrence of cholesteatoma.  Ref: 12533693 Lab Invest, 2003
- MATERIALS AND METHODS The K sam  [ **FGFR2** ]  gene encoding the receptor for keratinocyte growth factor  [ **FGF7** ]  KGF  [ **FGF7** ]  was transduced to three factor dependent hematopoietic cell lines Ba F 2Dcl3, and UT 7 GM using retroviral vector, and their proliferation, differentiation, and intracellular signaling were studied.  Ref: 11937263 Exp Hematol, 2002
- METHODS To quantify the expression of fibroblast growth factor receptor isoforms  **FGFR2**  IIIb,  **FGFR2**  IIIc, FGFR1 IIIc, and fibroblast growth factors FGF2 and  **FGF7**  in normal and tumoral human prostate tissues, and human prostatic epithelial cell lines, we used quantitative real time polymerase chain reaction.  Ref: 12111699 Prostate, 2002
- KGF  [ **FGF7** ]  and KGFR  [ **FGFR2** ]  expression was examined by immunohistochemistry using rabbit anti human KGF  [ **FGF7** ]  and anti human KGFR  [ **FGFR2** ]  polyclonal antisera raised in our laboratories against synthetic peptides corresponding to parts of human KGF  [ **FGF7** ]  and KGFR  [ **FGFR2** ] , respectively.  Ref: 12533693 Lab Invest, 2003
- ... neither one stimulated FGF 1 signaling via FGFR1 or FGF 7  [ **FGF7** ]  via  **FGFR2**  IIIb.  Ref: 11714710 J Biol Chem, 2002
- Parallel western blot analysis revealed that KGFRs were tyrosine phosphorylated both at early and late steps of internalization, suggesting that KGF  [ **FGF7** ]  and KGFR  [ **FGFR2** ]  remain associated in active complexes through the endocytic pathway.  Ref: 12122441 Histochem Cell Biol, 2002
- Epithelial KGF  [ **FGF7** ]  receptor KGFR  [ **FGFR2** ]  mRNA expression was studied using real time reverse transcriptase polymerase chain reaction RT PCR.  Ref: 12757114 JPEN J Parenter Enteral Nutr< MedlineTA>JPEN J Parenter Enteral Nutr, 2002
- In vivo inhibition of keratinocyte growth factor  [ **FGF7** ]  receptor  [ **FGFR2** ]  expression by estrogen and antagonism by progesterone in the mouse mammary gland.  Ref: 11691652 J Endocrinol, 2001
- Examination of hormonal regulation of the fibroblast growth factor FGF 7  [ **FGF7** ]   **FGFR2**  IIIb receptor system in the mammary gland reveals that mammogenic hormones differentially regulate the synthesis of stromal growth factors and their epithelial receptors.  Ref: 11897505 J Steroid Biochem Mol Biol, 2002
- Our results support the notion that the paracrine KGF  [ **FGF7** ]  KGFR  [ **FGFR2** ]  system in the skin can act in concert with the autocrine vitamin D system in keratinocytes to promote keratinocyte proliferation and survival under situations of stress and injury.  Ref: 12761878 J Cell Biochem, 2003
- Tumour cells expressed  **FGF7**  mRNA and protein, and low levels of  **FGFR2**  mRNA.  Ref: 12569015 Int J Radiat Biol, 2003
- UVB induced activation and internalization of keratinocyte growth factor  [ **FGF7** ]  receptor  [ **FGFR2** ] .  Ref: 12717419 Oncogene, 2003
- Further, we observed that the level of KGF  [ **FGF7** ]  receptor KGFR  [ **FGFR2** ]  expression in ER positive cells was much greater than in the ER negative cell lines.  Ref: 11688962 Clin Exp Metastasis, 2000
- In contrast, only relatively long oligosaccharides supported receptor binding and activation in the FGF 1.FGFR1 or FGF 7  [ **FGF7** ] . **FGFR2**  IIIb setting.  Ref: 11714710 J Biol Chem, 2002
- Murine brain capillary endothelial cells, denoted IBE cells, express small amounts of IIIc  **FGFR2** , which is not activated by keratinocyte growth factor  [ **FGF7** ]  KGF  [ **FGF7** ] .  Ref: 11735116 Biochem Biophys Res Commun, 2001
- KGF  [ **FGF7** ]  and KGFR  [ **FGFR2** ]  mRNA expression were increased after 14 28 days 5 fold and then returned to control levels.  Ref: 11737224 Eur J Clin Invest, 2001
- These peptide growth factors and their receptors c met and keratinocyte growth factor  [ **FGF7** ]  receptor  [ **FGFR2** ]  KGFR  [ **FGFR2** ]  are overexpressed in pancreatic cancer.  Ref: 11737224 Eur J Clin Invest, 2001
- Previously, FGF 7  [ **FGF7** ]  mRNA expression was reported in VSMC, but KGFR  [ **FGFR2** ]  mRNA was not detected.  Ref: 12608893 Pathol Int, 2003
- The effects of ethanol and acetone on the mRNA levels of genes characteristic for proliferating keratinocytes such as alpha5 integrin, keratinocyte growth factor  [ **FGF7** ]  receptor  [ **FGFR2** ]  and cyclin D1 were studied by reverse transcriptase polymerase chain reaction.  Ref: 12720008 Arch Dermatol Res< MedlineTA>Arch Dermatol Res, 2003
- In the present study, we investigated the possible involvement of KGF  [ **FGF7** ]  and its receptor, KGFR  [ **FGFR2** ] , in the pathogenesis of cholesteatoma using in situ hybridization and immunohistochemistry, respectively.  Ref: 12533693 Lab Invest, 2003
- The endocytic pathway followed by the keratinocyte growth factor  [ **FGF7** ]  receptor  [ **FGFR2** ] .  Ref: 12122441 Histochem Cell Biol, 2002
- Keratinocyte growth factor  [ **FGF7** ]  receptor  [ **FGFR2** ]  is up regulated in cyclosporin A induced gingival hyperplasia.  Ref: 12351666 J Dent Res, 2002
- To test this hypothesis, TGFbeta1, TGFbeta2 TGFbeta3, TGFbetaR I, TGFbetaR II, TGFalpha, EGF, EGFR, KGF  [ **FGF7** ]  and KGFR  [ **FGFR2** ]  gene and protein expression were analyzed in dorsal and ventral lobes of aging rat prostates,, 1 4, and 28 30 months.  Ref: 11906188 Biochem Biophys Res Commun, 2002
- The present study was therefore carried out to determine whether expression of the keratinocyte growth factor  [ **FGF7** ]  receptor  [ **FGFR2** ]  is up regulated in gingival hyperplasia compared with normal gingiva.  Ref: 12351666 J Dent Res, 2002
- MATERIALS AND METHODS In the present study three KGF  [ **FGF7** ]  inhibitors were employed Heparin, Innohep, a low molecular weight heparin LMWH and KGFR2 beta IIIb Fc, a chimeric KGFR  [ **FGFR2** ]  fragment.  Ref: 12529961 Anticancer Res< MedlineTA>Anticancer Res, 2002
- Assessment of the proliferative activity of cholesteatoma using the labeling index for Ki 67 showed a significantly higher Ki 67 labeling index 66% in KGF  [ **FGF7** ]  KGFR  [ **FGFR2** ]  cases than other cases.  Ref: 12533693 Lab Invest, 2003
- The expression of iNOS, KGF  [ **FGF7** ]  and KGFR  [ **FGFR2** ]  in laser wounds was more intense than in electrocautery wounds.  Ref: 11858949 J Dermatol Sci, 2002
- Therefore, the increase in the number of dividing cells and in the expression level of iNOS, KGF  [ **FGF7** ]  and KGFR  [ **FGFR2** ]  may induce earlier and thicker reepithelization in laser wounds than in electrocautery and scalpel wounds.  Ref: 11858949 J Dermatol Sci, 2002
- Expression of keratinocyte growth factor  [ **FGF7** ]  receptor  [ **FGFR2** ]  KGFR  [ **FGFR2** ]   **FGFR2**  IIIb in vascular smooth muscle cells.  Ref: 12608893 Pathol Int, 2003
- In epithelial tissues, keratinocyte growth factor  [ **FGF7** ]  KGF  [ **FGF7** ]  or FGF 7  [ **FGF7** ]  originates in the stroma while its receptor KGFR  [ **FGFR2** ]  or  **FGFR2**  IIIb is present only in the epithelium.  Ref: 11691652 J Endocrinol, 2001
- There was a significant correlation between KGF  [ **FGF7** ]  KGFR  [ **FGFR2** ]  expression and recurrence.  Ref: 12533693 Lab Invest, 2003
- The gene expression of HGF and keratinocyte growth factor  [ **FGF7** ]  KGF  [ **FGF7** ]  in gingival connective tissue, and c met, HGFA and KGF  [ **FGF7** ]  receptor KGFR  [ **FGFR2** ]  in gingival epithelial tissues was monitored using RT PCR.  Ref: 12613501 J Oral Sci, 2002
- Vitamin D enhances mitogenesis mediated by keratinocyte growth factor  [ **FGF7** ]  receptor  [ **FGFR2** ]  in keratinocytes.  Ref: 12761878 J Cell Biochem, 2003
- Thus, KGF  [ **FGF7** ] , KGFR  [ **FGFR2** ]  and or the KGF  [ **FGF7** ]  signaling pathway may be important therapeutic targets for the treatment or prevention of breast cancer metastasis.  Ref: 11688962 Clin Exp Metastasis, 2000
- Wounds were examined by transmission electron microscopy, the detection of free 3 OH DNA ends and immunohistochemistry of proliferating cell nuclear antigen PCNA, inducible nitric oxide synthase iNOS, keratinocyte growth factor  [ **FGF7** ]  KGF  [ **FGF7** ]  and keratinocyte growth factor  [ **FGF7** ]  receptor  [ **FGFR2** ]  KGFR  [ **FGFR2** ] .  Ref: 11858949 J Dermatol Sci, 2002
- The low  **FGFR2**  expression as well as the  **FGF7**  expression in the tumour cells may contribute to their resistance to rHuKGF treatment.  Ref: 12569015 Int J Radiat Biol, 2003
- The objective of this study was to assess the effect of repifermin on in vitro tumor cell proliferation and in vivo tumor growth using a variety of human carcinoma cell lines with differing growth rates and levels of KGF  [ **FGF7** ]  receptor KGFR  [ **FGFR2** ]  expression.  Ref: 12203102 Cancer Chemother Pharmacol, 2002
